# Sampling nucleotide diversity in cotton

**DOI:** 10.1186/1471-2229-9-125

**Published:** 2009-10-20

**Authors:** Allen Van Deynze, Kevin Stoffel, Mike Lee, Thea A Wilkins, Alexander Kozik, Roy G Cantrell, John Z Yu, Russel J Kohel, David M Stelly

**Affiliations:** 1 Seed Biotechnology Center, University of California, 1 Shields Ave, Davis, CA, USA; 2 Department of Plant and Soil Science, Texas Tech University, Experimental Sciences Building, Room 215, Mail Stop 3132, Lubbock, TX 79409-3132, USA; 3 Genome and Biomedical Sciences Facility, University of California, 1 Shields Ave, Davis, CA, USA; 4 Monsanto, 1 800 N. Lindbergh Blvd, St Louis, MO 63167, USA; 5 USDA-ARS, Southern Plains Agricultural Research Center, 2881 F&B Road, College Station, TX 77845, USA; 6 Department of Soil and Crop Sciences, Texas A & M University, College Station, TX 77843, USA

## Abstract

**Background:**

Cultivated cotton is an annual fiber crop derived mainly from two perennial species, *Gossypium hirsutum *L. or upland cotton, and *G. barbadense *L., extra long-staple fiber Pima or Egyptian cotton. These two cultivated species are among five allotetraploid species presumably derived monophyletically between *G. arboreum *and *G. raimondii*. Genomic-based approaches have been hindered by the limited variation within species. Yet, population-based methods are being used for genome-wide introgression of novel alleles from *G. mustelinum *and *G. tomentosum *into *G. hirsutum *using combinations of backcrossing, selfing, and inter-mating. Recombinant inbred line populations between genetics standards TM-1, (*G. hirsutum*) × 3-79 (*G. barbadense*) have been developed to allow high-density genetic mapping of traits.

**Results:**

This paper describes a strategy to efficiently characterize genomic variation (SNPs and indels) within and among cotton species. Over 1000 SNPs from 270 loci and 279 indels from 92 loci segregating in *G. hirsutum *and *G. barbadense *were genotyped across a standard panel of 24 lines, 16 of which are elite cotton breeding lines and 8 mapping parents of populations from six cotton species. Over 200 loci were genetically mapped in a core mapping population derived from TM-1 and 3-79 and in *G. hirsutum *breeding germplasm.

**Conclusion:**

In this research, SNP and indel diversity is characterized for 270 single-copy polymorphic loci in cotton. A strategy for SNP discovery is defined to pre-screen loci for copy number and polymorphism. Our data indicate that the A and D genomes in both diploid and tetraploid cotton remain distinct from each such that paralogs can be distinguished. This research provides mapped DNA markers for intra-specific crosses and introgression of exotic germplasm in cotton.

## Background

The cotton family consists of 45 diploid species (2n = 2x = 26) representing eight genome groups (A, B, C, D, E, F, G, K) and five AD allotetraploid species (2n = 4x = 52) that are inter-crossable to various degrees. Cultivated cotton is an annual fiber crop derived mainly from two perennial species, *Gossypium hirsutum *L. or upland cotton, and *G. barbadense *L., extra long-staple fiber Pima or Egyptian cotton. These two cultivated species are among five allotetraploid species presumably derived monophyletically from a single polyploidization event that occurred 1-2 MYA between ancestors most closely represented today by *G. arboreum *(A2 genome) and *G. raimondii *(D5 genome). Breeding of cotton is primarily focused on intra-specific crosses to introduce transgenic traits and to improve baseline lint yield and quality [1], although significant advances can be made through inter-specific introgression [2-4]. The relatively recent speciation of tetraploid cotton affords opportunities to transfer novel traits between species, but also amplifies the challenge of maintaining the high yields and quality requisite of commercial products. *G. tomentosum *(AD3) and *G. mustelinum *(AD4) are rich sources of novel traits that are currently being mined to improve cotton agronomy and fiber. DNA markers can provide means of detecting, manipulating and identifying genes associated with desirable agronomic and quality traits within breeding programs, as well as novel alleles from wide crosses. They have shown to be useful in accelerating the transfer of novel traits into elite backgrounds, particularly when these markers have been placed on genetic maps [5].

The most extensive genetic maps in cotton have been derived mainly from wide crosses between the two AD-genome species *G. hirsutum *and *G. barbadense *[6-8]. Inter-specific maps also exist between *G. hirsutum *and *G. tomentosum *[9]. DNA markers have been oriented to chromosomes and used to establish co-linearity among genomes and species using radiation hybrids and hypo-aneuploid F_1 _hybrids, available for most chromosomes. In total, approximately 5,000 DNA markers have been mapped. These were derived from approximately 3,300 restriction fragment length polymorphisms (RFLPs), 700 amplified fragment length polymorphisms (AFLPs), 1,000 simple sequence repeats (SSRs), and 100 single nucleotide polymorphisms (SNPs) [10]. Furthermore, 2,584 sequence-tagged site (STS) loci are mapped in an AD genome and 1,014 in and the D genome [8,11]. An EST-SSR map with 1,017 loci is also available [12]. Only a few low resolution intra-specific maps that focus on specific traits exist due to the low level of polymorphism within a species and the paucity of cost-efficient markers available to be used in breeding programs.

The vast majority of markers in cotton that are useful in breeding are as SSRs. Over 8,000 pairs of SSR primers are identified in cotton from *G. arboreum, G. raimondii *and *G. hirsutum *[13]. The frequency of polymorphism within species has been reported to be limited to 11% [14]. Informative, abundant, high-throughput markers associated with genes such as SNPs or insertion/deletions (indels) are desirable for both breeding and genetic analyses. Expressed genes are available as templates to study variation. In, *G. arboreum*, 24,597 non-redundant transcripts are available; with 27,355 in *G. raimondii; *and 63,138 in *G. hirsutum *[15]. The goal of the current project was to design a strategy to efficiently identify and characterize SNP markers that are useful to manipulate and transfer novel alleles to breeding germplasm in cotton. Different DNA templates were evaluated for their specificity to amplify single-copy loci, and polymorphism within and among species, with emphasis on cultivated cotton. We show that single-copy loci can be efficiently amplified in cotton despite redundancy conferred by its allopolyploid origin, and that they can be mapped to specific genomes. The information can be queried in the Cotton Marker Database which has been modified for presentation of SNP data [16]

## Results and Discussion

### Defining optimum regions to sample for SNPs in cotton

Our goal was to define and optimize a SNP discovery strategy in cotton that exploits current genomic resources. The disomic polyploid nature of cotton poses a particular challenge in that most loci are duplicated, and breeding germplasm is derived from a relatively narrow genetic base [17,18]. A re-sequencing strategy developed by our lab was modified to anchor and screen DNA primer pairs that amplify single-copy, polymorphic regions of the genome relevant to current breeding germplasm that can also be used for introgression of novel alleles from exotic germplasm (See Materials and Methods and [19]). To address this, we empirically evaluated the proportion of single-copy sequences obtained from amplicons originating from different genomic regions, namely: BAC-end sequences, sequences flanking SSRs or predicted introns in ESTs, and sequences in the 3' or 5' untranslated region of ESTs (Table 1). Although the majority (93%, data not shown) of amplifiable primer sets showed single bands on agarose gels, SSCP analysis revealed that only 51% and 40% of the primer pairs amplified single-copy sequences in *G. arboreum *and *G. hirsutum*, respectively. With SSCP analysis of single varieties, an amplicon from a single-copy locus in homozygous state, whether from a diploid or tetraploid is expected to display only two bands, one from sense and one anti-sense strands. As the lines being assayed were near-homozygous, only loci with two SSCP bands were carried forward.

**Table 1:** Summary statistics for SNPs and indels among 24 lines of cotton derived from different DNA templates.

**DNA template**	**Primer pairs designed**	**Amplified primer pairs (%)**	**Single-copy loci (%)^1^**	**Polymorphic SNP loci (%)^2^**	**SNPs**	**SNPs/locus^1^**	**bp/SNP^2,3^**	**Polymorphic indel** **loci (%)^2^**	**Indels**	**Indels/locus**	**bp/indel^2,3^**
EST 3' end	160	102 (64)	45 (28,44)	30 (67)	144	3.2	252	14 (31)	49	1.1	741
EST 5' end	988	802 (73)	417 (42,52)	142 (34)	417	1.0	807	37 (9)	109	0.3	3,087
COS	576	523 (91)	201 (35,38)	56 (28)	296	1.5	548	20 (10)	70	0.3	2,317
Intron	126	98 (78)	44 (35,45)	17(39)	46	1.0	772	11 (25)	32	0.7	1,110
GSP	48	29 (60)	23 (48,79)	11 (48)	40	1.7	464	5 (22)	12	0.5	1,547
SSR	52	46 (88)	21 (40, 46)	14 (67)	62	3.0	273	5 (24)	7	0.3	2,421
BAC	24	7(29)	0 (0,0)	n/a	n/a	n/a	n/a	n/a	n/a	n/a	n/a

Total	1,974	1,607 (81)	751 (47)	270 (36)	1,005	1.3	603	92 (12)	279	0.4	2,172

^1^First number is percent of designed; second is percent of amplified

^2^Relative to single-copy loci

^3^Adjusted to average contig length of 807 bp

The amplicon templates exhibited a range of amplification, copy number and polymorphism (Table 1). The low rate of amplification (29%) achieved when primers were designed from BAC-end genomic sequence deterred us from using this as template for primer design. The relatively poor rate of success might have resulted from inadequate quality of BAC-end genomic sequence available at the time (Sept, 2004). Cotton genome-specific (GSP) amplicons, those derived from primers spanning a SNP between *G. arboreum *and *G. raimondii*, resulted in the highest percentage (48%) of single-copy sequences (Table 1). Primer pairs neighboring genomic SSRs also resulted in a high percentage (46%) of single-copy sequences, but the relatively small amount of sequence flanking each SSR, 150-200 bp, limited the number of nucleotides available within an amplicon for SNP discovery. Amplicons that encompass introns or are derived from a conserved orthologous set (COS) of sequences that are single copy in *Arabidopsis *and cotton ESTs showed excellent amplification. However, as their primers are anchored in coding regions, they are likely to be the most conserved across paralogs, as indicated by the relatively low proportion (38% for introns and 45% for COS primers) of single-copy sequences. The 5' UTR of ESTs are the most abundant in the EST dataset and resulted in the highest proportion of amplicons with single-copy loci overall (42%), next to the GSPs that became available only late in the project. It is important to note that the ESTs used were created by capturing sequences from the 3' UTR and sequencing from either the 3' or 5' end. Consequently, the 5' sequences may not necessarily represent the 5' terminus of genes if clones were not full length. As a comparison, Chee et al. (2004) used sequences from *G. arboreum *to amplify *G. hirsutum *[20]. The authors reported that 33% (16/89) of primer pairs yielded amplicons from single-copy loci, which is similar to the current results (average 38% across EST-derived amplicons, Table 1). This indicates the high level of homology of exons between homoeologous genomes in tetraploid cotton. The results of this study indicate that although genomic regions harbor genetic diversity, strategies must be developed to ensure that allelic diversity and not diversity between homoeologs and paralogs are being assayed.

### Diversity of genomic regions

To study the diversity of cotton, we tested different DNA templates to select the optimum regions that will yield single-copy, yet polymorphic amplicons from PCR. Of the 1,974 primer pairs designed, 8% were from the 3' end; 50% 5'end; 29% COS; 6% intron; 3% SSR; 2% genome-specific primers; and 1% genomic (BAC-end) primers. Eighty-one percent successfully amplified DNA in a single *G. arboreum *and *G. hirsutum *line and 47% produced single-copy amplicons based on SSCP gels (Table 2). The relatively high amplification rate across species from primers designed mainly from diploid species confirms the transferability of markers across species as indicated in previous studies [21-25]. To pre-screen primer pairs for polymorphism, DNA pools representing increasing diversity within *G. hirsutum *and *G*. *barbadense *germplasm and among these two species were amplified and sequenced. Pool 1 represents *G. hirsutum*; Pool 2, *G. hirsutum/G. hirsutum *race *yucatenense*; and Pool 3, *G. hirsutum *and *G. barbadense *(Table 2). Polymorphism within pools was identified as heterozygotes, whereas polymorphism among pools was identified as differences in homozygous alleles. Polymorphic loci were then sequenced individually in the forward and reverse directions in 24 lines (Table 2). Forty-nine percent of primers with single-copy loci were polymorphic within or among pools. Overall, the pools represent the two cultivated species in the United States, *G. hirsutum *and *G. barbadense*, which explains the high polymorphism (percent polymorphic loci) and SNP frequency (bp/SNP) compared to within species polymorphism (Tables 2, 3 and 4). The SSCP pre-screening and pooling strategy saved 77% of the resources compared to direct sequencing of individuals with 1607 amplifiable primers (Table 5).

**Table 2:** Germplasm panel sequenced for SNP or indel discovery.

**Line^1^**	**Genome**	**Description**	**CMD panel**	**Pool^2^**
Acala Maxxa	[AD]_1_	California Upland cotton and BAC donor	Yes	Pool 2, 3
AHA 6-1-4	[AD]_1_	Upland cotton		
Auburn 623RNR	[AD]_1_	Upland cotton		
Coker 312	[AD]_1_	Upland cotton		
Deltatype Webber	[AD]_1_	Upland cotton		
DPL 458BR	[AD]_1_	Upland cotton	Yes	Pool 1
Fibermax 832	[AD]_1_	Upland cotton	Yes	Pool 1
Paymaster 1218BR	[AD]_1_	Upland cotton	Yes	
PD-1	[AD]_1_	Upland cotton		Pool 2
Sealand 542	[AD]_1_	Upland cotton		Pool 1
Stoneville 20	[AD]_1_	Upland cotton		
Stoneville 4892BR	[AD]_1_	Upland cotton	Yes	Pool 1
Tamcot Sphinx	[AD]_1_	Upland cotton		Pool 2
Tidewater Seabrooks	[AD]_1_	Upland cotton		
TM-1	[AD]_1_	Genetic standard (BAC donor/RI parent)	Yes	Pool 3
Wilt Acala 1517	[AD]_1_	California Upland cotton		
TX 2094	[AD]_1_	*G. hirsutum *race *yucatanense*		Pool 2

3-79	[AD]_2_	Genetic standard (fiber QTLs/RI parent)	Yes	Pool 3
Pima S-6	[AD]_2_	Pima germplasm breeding source	Yes	
Pima S-7	[AD]_2_	Pima germplasm breeding source		Pool 3

*G. tomentosum*	[AD]_3_	Introgression breeding source	Yes	
*G. mustelinum*	[AD]_4_	Introgression breeding source	Yes	
*G. arboreum*	A_2-8_	A-genome species representative	Yes	
*G. raimondii*	D_5-3_	D-genome species representative	Yes	

^1^All 12 members of the Cotton Marker Database Panel were included [13]

^2^Pools of 4 lines representing cotton germplasm were used to screen primer sets (see Materials and Methods)

**Table 3:** Species-specific statistics for *Gossypium *SNPs and indels.

**Species**	**N**	**Loci with SNPs**	**Number of SNPs**	**Loci with SNPs (%)^1^**	**SNPs/locus^1^**	**bp/SNP^1,2^**	**Loci with indels**	**Number of indels**	**Loci with indels (%)^1^**	**Indels/locus^1^**	**Bases/indel^1,2^**
*G. arboreum*	1	149	238	19.8	0.3	2,546	34	67	2.1	0.1	9,046
*G. raimondii*	1	142	379	18.9	0.5	1,599	29	66	1.8	0.1	9,183
*G. hirsutum*^3^	16	124	245	16.5	0.3	2,474	70	161	4.4	0.2	3,764
*G. barbadense*	3	208	439	27.7	0.6	1,381	48	117	3.0	0.2	5,180
*G. mustelinum*	1	182	432	24.2	0.6	1,403	42	94	2.6	0.1	6,447
*G. tomentosum*	1	156	382	20.8	0.5	1,587	34	84	2.1	0.1	7,215

Total	24	270	1,005	36.0	1.3	603	92	279	5.8	0.4	2,172

^1^Relative to 751 single-copy loci

^2^Adjusted to average contig length of 807 bp

^3^Excluding *G. hirsutum *race *yucatenense*

**Table 4:** Summary statistics for SNPs and indels between pairs of *Gossypium *species. The percentage is calculated out of total of 1005 SNPs and 279 indels.

**Number of SNPs**	*Gr*^1^	*Gh*	*Gb*	*Gm*	*Gt*	**Number of indels**	*Gr*	*Gh*	*Gb*	*Gm*	*Gt*
	
*G. arboreum*	*317*	*303*	*201*	*334*	*261*	*G. arboreum*	*111*	*140*	*122*	*93*	*94*
*G. raimondii*		*452*	*430*	*266*	*317*	*G. raimondii*		*169*	*153*	*84*	*115*
*G. hirsutum*			*396*	*427*	*451*	*G. hirsutum*			*140*	*92*	*133*
*G. barbadense*				*396*	*448*	*G. barbadense*				*91*	*113*
*G. mustelinum*					*410*	*G. mustelinum*					*96*
	
**SNP Polymorphism (%)**	*Gr*	*Gh*	*Gb*	*Gm*	*Gt*	**Indel Polymorphism (%)**	*Gr*	*Gh*	*Gb*	*Gm*	*Gt*
	
*G. arboreum*	32	30	20	33	26	*G. arboreum*	40	50	44	33	34
*G. raimondii*		45	43	26	32	*G. raimondii*		61	55	3	41
*G. hirsutum*			39	42	45	*G. hirsutum*			50	33	48
*G. barbadense*				39	45	*G. barbadense*				33	41
*G. mustelinum*					41	*G. mustelinum*					34

^1 ^Gr = G. raimondii, Gh = G. hirsutum, Gb = G. barbadense, Gm = G. mustelinum, Gt = G. tomentosum

**Table 5:** Summary of results for primer pairs tested in pools.

**Primer results^1^**	**Number of primers**	**Percentage of preceding primer pool**	**Percentage of all primers tested**
Tested	1974	-	-
Amplified	1607	81	81
Single-copy	751	47	38
Polymorphic in pools	365	49	18
Successfully sequenced	351	96	18

^1^Thirty-four single-copy loci were sequenced without pre-screening in pools.

Only amplicons showing polymorphism in pools were sequenced in individual lines. The germplasm panel included 16 *G. hirsutum *and three *G. barbadense *genotypes, plus tetraploids *G. mustelinum *and *G. tomentosum *and diploids *G. arboreum *and *G. raimondii *(Table 2). Sequencing of DNA templates resulted in different frequencies of SNPs and indels (Table 1). Sequences from 3' UTRs (252 bp/SNP), and SSR-associated (273 bp/SNP) sequences yielded 2-3 fold the frequency of SNPs than 5'ends, COS, GSP and introns (464-807 bp/SNP). It is important to note that COS and GSP sequences contained introns. Similarly the frequency of indels was least in 5' end sequences and greatest in 3' UTRs (Table 1). These results are consistent with those in maize showing that 3' UTRs are a rich source of nucleotide diversity [26,27]. The lowest diversity in the 5'ends is consistent with representation of more highly conserved coding sequences [19] . In comparison, the vacuolar H^+^-ATPase subunit [28] and Myb transcription factor families [29] were examined for diversity in diploid and tetraploid cotton species. The, 3' UTRs were 10-fold more polymorphic than coding sequences for Myb transcription factors [29].

Alternatively, designing primers from conserved single-copy sequences (COS), or genome-specific primers (GSP) is an efficient method to access variation in introns for single loci in cotton. A closer examination at the different strategies indicates that GSP-based primers are less likely to amplify informative products than COS (60 vs. 91%), but more likely to target single-locus sequences when they do (79 vs. 45%). The majority of the COS sequences in our dataset were derived from diploid progenitors (see Materials and Methods). A COS-derived from tetraploid cotton only is likely to yield a larger proportion of single-copy loci in tetraploids., Cotton intron regions were found to be less conserved than exons in the H^+^-ATPase subunit family [28]. Intron sequences have shown to be 3.7-fold more polymorphic than exons in cotton [20]. COS-based amplicons have also yielded a very high proportion of single-copy sequences (> 95%) in Solanaceae [19,30].

Another template target is to use predicted SNPs between diploid progenitor species to anchor primers to specific genomes (GSPs). Yang et al. [31] predicted 32,229 genome-specific SNPs from EST databases with 31% showing perfect concordance to the A or D genomes in the genotypes examined. In the current study, 3,000 SNPs were identified between only the diploid species and not within the tetraploid species (data not shown). As we sampled introns, many of these would be novel to EST-mined SNPs. Only 317 SNPs between *G. arboreum *and *G. raimondii *(Table 4) are polymorphic in *G. hirsutum *and *G. barbadense*. In our study, the estimates of diversity within and among species may not be representative of other species than *G. hirsutum *and *G. barbadense*, as only sequences that were polymorphic within and among these species were characterized. To ensure that single copy loci were being assayed, only SNPs that had clear homozygotes in at least one tetraploid were called.

### Diversity of cotton germplasm

#### Within species diversity

SNPs were recorded as a base substitution relative to the consensus sequence derived from all genotypes. Although the number of individuals sampled varied, the species-specific diversity (as measured by the number of bases per SNP) was similar with two-fold lower frequency of SNPs for *G. arboreum *and *G. hirsutum *than the other species tested (Table 3, Additional File 1, Table S1). *G. barbadense *showed the highest level of diversity of the species tested with 1 SNP per 1381 bases and 28% of its loci being polymorphic. Conversely, *G. hirsutum *has the highest frequency of indels and the diploid progenitor species the least (Table 3, and Additional File 1, Tables S1 and S2). The average frequency of SNPs among 16 *G. hirsutum *lines was 1/2474 bp (0.04%). In Adh genes, within *G. hirsutum *and *G. barbadense *diversity ranged from 1 to 3 SNPs in 983 bases (0.1-0.3%) [32]. With the few reports of SNP frequencies in cotton, within *G. hirsutum *(3-4 lines), SNP frequency ranged from 0/1,000 bp [29,32], 1/5,000 bp (0.02%) [22] to 1/947 bp (1%) [21] in Adh, Myb and expansin genes, respectively. Estimates of SNP divergence within and among species in the referenced studies were greater than those reported in our study for over 1,900 loci.

#### Among species diversity

SNP diversity in cotton has a large range that is both genome and gene specific. Compared to *G. hirsutum*, SNP frequency in *G. arboreum, G. barbadense, G. mustelinum, G. tomentosum and G. raimondii *ranged from 1/2,000 bp (0.05%) to 1/1,341 bp/SNP (0.075%) in order of increasing divergence (Table 4). Estimates among these species ranged from 1/51 bp per SNP (1.96%) in Myb and expansin genes between *G. raimondii *and *G. hirsutum *[22,29] to 1/714 bp per SNP (0.14%) between *G. hirsutum *and *G. barbadense *in R2R3 Myb transcription factors [21,33]. The discrepancy in estimates is likely due to gene-specific estimates, number of genes, germplasm used and the regions of the genes being sampled (coding vs. non coding). Non-coding sequence was reported to be 2-3 fold more polymorphic than coding regions [21,22].

Comparisons of tetraploid species with *G. raimondii *(D-genome) were consistently more divergent than those with *G. arboreum *(A-genome) except for *G. mustelinum *(Table 4). This agrees with observations using SSRs. EST-derived SSRs from diploid species tended to map more often to their orthologous genomes in corresponding tetraploid species [23], with *G. raimondii*-derived SSRs (43% polymorphism) being more polymorphic than *G. arboreum *derived SSRs (18%) in the same cross [24,34]. Furthermore, in comparisons between A-genome diploids, a D-genome diploid, and tetraploids (*G. hirsutum *and *G. barbadense*), the rate of divergence in 48 genes was significantly higher in D-genomes than A-genomes, although the rate of divergence was gene-specific [33]. This was verified for specific gene families of transcription factors, Adh and expansins [21,22,32] in cotton.

Several methods have been proposed for SNP discovery in allopolyploids and highly duplicated genomes including *in silico *analysis [31,35], genome specific-PCR [36], cloning and sequencing [29]. We have evaluated genome-specific amplification and high-throughput direct sequencing using M13-tailed amplicons combined with targeted primer design and pre-screening of primer pairs. We show that several options are feasible for high-throughput SNP discovery in cotton, each with their own advantages and disadvantages. The current research agrees with current literature that although tetraploid cottons were derived from a single polyploidization event from their diploid progenitors only 1-2 MYA [37], the genomes remain distinct and have sufficient diversity for breeding.

### Linkage Mapping

**Figure 1:**
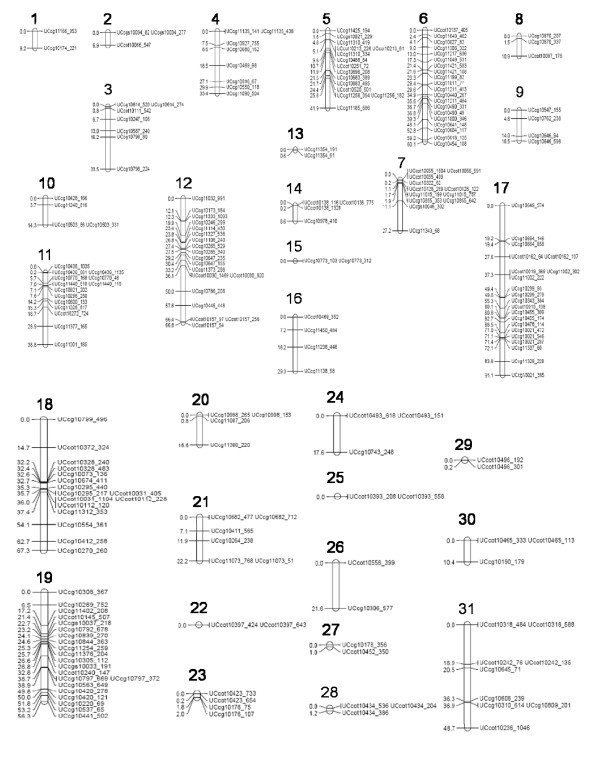
**Genetic map of 223 SNP markers in 186 recombinant inbred lines from a cross between TM-1 and 3-79**. Genetic distances in cM [46].

SNPs were evaluated as markers by designing a 384-SNP array for the Illumina Golden Gate assay^®^. Of the 384 SNPs on the array, 268 putative SNPs representing 240 contigs were expected to be polymorphic between the parents of our mapping population, TM-1 (*G. hirsutum*) and 3-79 (*G. barbadense*) and validated by assessing their segregation and amenability to linkage mapping. Of the 268 expected parental SNPs, 247 polymorphisms were detected using the Illumina assays on a population of 186 recombinant inbred lines (RILs) [23]. Segregation was as expected (1:1) for 188 markers, whereas 59 had skewed segregation resulting in 223 SNPs being placed on the linkage map (Figure 1). Markers that were not mapped had missing data or skewed segregation. The SNP markers are added to the TM-1/3-79 base map (Figure 1, and Yu et al in preparation) providing new tools for high-throughput genotyping in cotton. The SNP data are thus cross-referenced to several genetic [6,8,14] and physical maps [38,39] via common SSRs (Yu et al in preparation). All SNPs, indels and flanking sequences can be accessed through the CMD database[16].

Although markers were evenly distributed within linkage groups, there was a disproportionate number of markers on linkage groups in the A subgenome (191 markers, 86%) vs. the D subgenome (32 markers, 14%; Figure 1, data not shown). At least 70% of the primers including those designed from 3'-end, 5'-end, intron and GSP, were derived exclusively from *G. arboreum *sequences, whereas the remainder were from *G. arboreum, G. hirsutum *and *G. raimondii *assemblies (see Materials and Methods). The fact that the majority of loci were derived from *G. arboreum *sequences and that these loci were in turn mapped to A-subgenome chromosomes in the TM-1 × 3-79 cross suggests that a) our approach for identifying single-locus markers was very effective; b) there is strong sequence conservation between the A_2 _genome of *G. arboreum*, and the A subgenomes of *G. hirsutum *([AD]_1_) and *G. barbadense *([AD]_2_), c) the A and D subgenomes of contemporary AD genomes are distinct from each other; and d) the A subgenomes of *G. hirsutum *and *G. barbadense *are moderately divergent. The above conclusions are emphasized in the present study first by amplifying and sequencing single-copy loci and being able to assay the same sequences using an independent assay, Illumina Golden Gate. Because our template sequences were primarily drawn from *G. arboreum *and selected for both PCR function and single-locus attributes using SSCP, it is quite possible that the populations of selected loci in the two subgenomes would have been differentially affected. The potential for bias across the two subgenomes precludes using these amplicon sequence or genotyping data to infer relative diversity of the A versus D subgenomes of *G. hirsutum *and *G. barbadense*. Our findings are not discordant with the theory that these two extant tetraploids originated from a common allopolyploid ancestor and have evolved independently long enough to create the detected variation and a significant degree of diploidization.

### The use of SNPs in cotton

SNP diversity depends on population size sampled and the natural evolutionary and directed selection within those populations. The present study indicates that a moderate amount of variation associated with genes exists in breeding germplasm (1 SNP in 2,474 bp in *G. hirsutum*) at the nucleotide level. *G. barbadense *germplasm sampled was 1.8 times as diverse (1 SNP in 1,381 bp) as *G. hirsutum *even though less than one fifth as many *G. barbadense *lines were sampled. The A and D genomes of tetraploid cotton are significantly divergent such that individual SNP loci can be assayed with high specificity. Although the above strategy for SNP identification and validation was fruitful, new high-throughput sequencing technologies such as 454 (Roche Biosciences, Branford, USA) Illumina Genome Analyzer (Hayward, USA) and SOLiD (Applied Biosystems, Foster City, USA) offer an opportunity to complement the current strategy to rapidly uncover nucleotide diversity at the whole genome level in multiple breeding lines. The present study demonstrates that sufficient SNP diversity exists in tetraploid cotton populations for genetic and breeding studies and it can be efficiently assayed.

## Conclusion

In this research, SNP and indel diversity is characterized for 270 single-copy polymorphic loci in cotton. A strategy for SNP discovery is described to pre-screen loci for copy number and polymorphism. Diversity was characterized in a broad set of breeding lines and exotic lines representing a standard germplasm panel indicating that *G. barbadense *is much more diverse than *G. hirsutum*. Our data also indicate that the A and D genomes in both diploid and tetraploid cotton remain distinct from each other such that homoeologs can be distinguished. All marker data and flanking sequences have been submitted to Cotton Marker database 

## Methods

### Plant materials

The 24 cotton lines and species screened for this study were chosen based on an expansion of the Cotton Marker Database (Table 2, [13]) standard germplasm panel assembled to represent the breadth of US cotton breeding germplasm and genetic standards. Consequently, the specific polymorphisms are expected to be relevant to these applications. The CMD panel has been genotyped with thousands of Simple Sequence Repeat (SSR) markers and serves as a resource to assess the utility of genetic markers in cotton. To address the low polymorphism in cotton breeding germplasm (11%) [14] the CMD panel was expanded to include 12 additional elite breeding lines (Table 1). The panel contains crossing parents from *G. tomentosum *and *G. mustelinum*, and representation of the diploid species, *G. arboreum *and *G. raimondii*. As our main goal is to develop markers relevant to breeding germplasm 16 *G. hirsutum *and three *G. barbadense *(Pima) lines were selected. An additional landrace, TX2094, was added to represent *G. hirsutum *race *yucatanense *[40]. All accessions represent self-pollinating or inbred lines, thus should be homozygous for the majority of the loci.

### Primer design and screening

To determine the optimum target template to identify SNPs specific to single-copy sequences in tetraploid cotton, we tested primer pairs from available BAC-end sequences; sequences flanking SSRs or predicted introns in ESTs; and sequences in the 3' UTR or 5' terminus of ESTs (Table 1). All sequences were downloaded from GenBank (Sept, 2004) except for SSR sequences kindly provided by Dr. Ben Burr, Brookhaven National Laboratories, NY). Based on the results, a database of primer sets with predicted product sizes of 600-800 bp was generated using Primer3 [41] to design primer pairs in the 3' or 5' termini of unigenes from *G. arboreum *ESTs [42]. As more ESTs became available, *G. arboreum *and *G. raimondii *ESTs were trimmed and assembled into 7,666 contigs with SNPs and deletions between these genomes. To evaluate the usefulness of cotton genome-specific primers, 48 primer pairs (GSP) were tested with a deletion in the forward primer and 48 with a SNP at the 3' end of the forward primer. A Conserved Orthologous Set for cotton of 2,390 contigs based on 27,878 *G. arboreum*, 35,509 *G. raimondii *and 14,354 *G. hirsutum *ESTs using the procedures described in Van Deynze et al (2007, [19]) was also generated as template for SNP discovery. A set of 576 primers (labeled with prefix of COT) was designed to amplify across predicted intron sites (based on Arabidopsis) with primers positioned 50-100 bp from the predicted introns

Two near-homozygous lines (*G. arboreum *and *G. hirsutum *breeding lines) were amplified and tested on agarose gels for amplification (Table 5). The amplified products were subsequently run on SSCP gels to screen for the presence of duplicated loci in amplification products. SSCP is a highly sensitive technique that detects variation in the nucleotide sequences of single-stranded molecules. Single-copy loci display two bands (the sense and antisense DNA strands). Loci displaying greater than two bands were not sequenced and were assumed to be not from a single locus. Primer pairs that successfully amplified a product and showed SSCP patterns of single-copy loci were tested for polymorphism using sequencing in a series of three pools representing different degrees of diversity in breeding germplasm: within elite *G. hirsutum*, a genetically diverse *G. hirsutum*, and between *G. hirsutum *and *G. barbadense *[19]. Each pool had three similar lines and one complementary genetically-distant line to maximize the chance of detecting a polymorphism within or among pools. Using a series of empirical tests with lines with known SNPs in ratios of 1:7, 1:5, 1:3 and 1:1, we determined that an unknown polymorphism can be reliably detected with sequencing with a 1:3 dilution. Pool 1 consisted of DPL458BR, Fibermax 832, Stoneville 4892BR, Sealand 5 42; Pool 2 consisted of PD1, Maxxa, Tamcot Sphinx, TX2094; and Pool 3 consisted of TM-1, 3-79, Pima S-7, Maxxa (Table 2). These pools represent increasing diversity in breeding germplasm. DNA was extracted from each line using Qiagen DNEASY (Qiagen, Valencia, USA) and was combined in equi-molar concentrations.

For all sequencing reactions, forward and reverse primers were tailed with M13 sequences and sequenced using standard protocols for Sanger sequencing (Applied Biosystems, Foster City, CA) in forward and reverse directions using a ABI 3730 (Applied Biosystems, Foster City, CA). Trace files were trimmed with Phred options -trim_cutoff 0.02" which translates to Phred 17 score [43]. Assembly was achieved with Phrap/Consed and options were set at "-retainduplicates and -forcelevel 5". These options were optimized to give the best trim and assembly parameters for calling SNPs. Stringent trim parameters are favored in this case to minimize the high number of false SNPs associated with poor sequence on the ends.

SNPs were first identified semi-manually using Polyphred as heterozygotes within pools or homozygous differences among pools. The line, HS200 (*G. hirsutum*), was used as reference to confirm that amplicons of single-copy loci were represented. Amplicons with putative SNPs were then amplified in the individual 24 lines (Table 2) and sequenced as described above. Only SNPs showing both homozygous alleles were called. Data was extracted from Polyphred using custom scripts ([44] See Supplemental files). Similarly, data for indels were extracted from Polyphred. Total polymorphism was calculated among genotypes and species as total number of SNPs, bases per SNP (SNP frequency) and percent polymorphic loci. Sequences are available through the Cotton Marker database [16].

A set of 384 SNPs was selected to develop an Illumina Golden Gate^® ^oligonucleotide pooled assay. In order of priority, SNPs were selected to maximize the number of loci represented that were polymorphic between TM-1 and 3-79, or were polymorphic within *G*. *hirsutum *with moderate minor allele frequencies (> 15%) in *G. hirsutum *germplasm sequenced (Table 2). SNPs were genotyped in 186 RILs, the parents, and the F_1 _as per manufacturer protocols at the University of California, Genome and Biosciences Facility, Davis, CA. Data were extracted and exported and mapped using JoinMap 4.0 [45] with the Kosambi mapping function[46]. A LOD score of at least 6.0 was used to determine the linkage groups, of which the marker orders were verified at LOD score 3.0. Individual linkage groups were assigned to respective chromosomes by use of the TM-1 × 3-79 base map (Yu et al, in preparation).

## List of abbreviations

BAC: bacterial artificial chromosome; COS: conserved orthologous set; EST: expressed sequence tag; Indel: insertion/deletion; SNP: single nucleotide polymorphism

## Authors' contributions

AV conceived, supervised and wrote the manuscript; KS carried out the research, contributed to methods and research and edited the paper; MK carried out the research, contributed to methods and research; AK helped conceive, analyse and edit the paper; TW aided in initiating the research, the EST database and edited the paper; RC inititated the research and developed the germplasm panel; JY contributed the mapping population, analysed the mapping data and edited the paper; RK contributed to the mapping population and analaysis; DS conceived the research, developed the germplasm panel, analyzed the data and edited the paper.

All authors have read and contributed to the writing of the manuscript.

## Supplementary Material

Additional File 1 **Supplementary tables**. **Table S1**. SNPs and associated sequence information. This worksheet describes the SNPs and the sequences they are derived from. **Table S2**. Indels and associated sequence information. This worksheet describes the SNPs and the sequences they are derived from. **Table S3**. Mapping data for TM-1 × 3-79 RIL population. This worksheet contains the data used for the mapping the SNPs. **Table S4**. Amplification and sequencing conditions used in this research. This worksheet describes the PCR protocol used to amplify and sequence genomic DNA. Click here for file
